# The neurotrophic hepatocyte growth factor attenuates CD8^+^ cytotoxic T-lymphocyte activity

**DOI:** 10.1186/1742-2094-10-154

**Published:** 2013-12-17

**Authors:** Mahdia Benkhoucha, Nicolas Molnarfi, Gregory Schneiter, Paul R Walker, Patrice H Lalive

**Affiliations:** 1 Department of Pathology and Immunology, Faculty of Medicine, University of Geneva, 1211 Geneva, Switzerland; 2 Department of Clinical Neurosciences, Division of Neurology, Unit of Neuroimmunology and Multiple Sclerosis, Geneva University Hospital and Faculty of Medicine, Gabrielle-Perret-Gentil 4, 1211 Geneva 14, Switzerland; 3 Centre of Oncology, Geneva University Hospitals and University of Geneva, 1211 Geneva, Switzerland; 4 Department of Genetic and Laboratory Medicine, Division of Laboratory Medicine, Geneva University Hospital, 1211 Geneva, Switzerland

**Keywords:** Hepatocyte growth factor (HGF), Cytotoxic T lymphocytes (CTL), Immune modulation, Central nervous system autoimmunity, Dendritic cells, Multiple sclerosis (MS), Immunological tolerance

## Abstract

**Background:**

Accumulating evidence suggests a deleterious role for CD8^+^ T cells in multiple sclerosis (MS) pathogenesis. We have recently reported that hepatocyte growth factor (HGF), a potent neuroprotective factor, limits CD4^+^ T cell-mediated autoimmune neuroinflammation by promoting tolerogenic dendritic cells (DCs) and subsequently regulatory T cells. Whether HGF modulates cell-mediated immunity driven by MHC class I-restricted CD8^+^ T cells remains to be determined.

**Methods:**

Here we examined whether HGF regulates antigen-specific CD8^+^ T cell responses using an established model of murine cytotoxic T lymphocyte (CTL)-mediated killing.

**Results:**

We found that HGF treatment of gp100-pulsed DCs reduced the activation of gp100-specific T cell receptor (Pmel-1) CD8^+^ T cells and subsequent MHC class I-restricted CTL-mediated cytolysis of gp100-pulsed target cells. The levels of perforin, granzyme B, IFN-γ, and the degranulation marker CD107a as well as Fas ligand were decreased among CD8^+^ T cells, suggestive of a dual inhibitory effect of HGF on the perforin/granzyme B- and Fas-based lytic pathways in cell-mediated cytotoxicity. Treatment of CD8^+^ T cells with concanamycin A, a potent inhibitor of the perforin-mediated cytotoxic pathway, abrogated CTL cytotoxicity indicating that blockade of the perforin-dependent killing is a major mechanism by which HGF diminished cytolysis of gp100-pulsed target cells. Moreover, HGF suppressed the generation of effector memory CTLs.

**Conclusions:**

Our findings indicate that HGF treatment limits both the generation and activity of effector CTL from naïve CD8^+^ T cells. Complementary to its impact on CD4^+^ T-cell CNS autoimmunity and myelin repair, our findings further suggest that HGF treatment could be exploited to control CD8^+^ T-cell-mediated, MHC I-restricted autoimmune dysfunctions such as MS.

## Background

Multiple sclerosis (MS), a chronic autoimmune disorder characterized pathologically by central nervous system (CNS) inflammation, demyelination, and axonal damage, has been traditionally attributed to self-reactive CD4^+^ T lymphocytes that escape tolerance [1]. Growing evidence, however, indicates that autoreactive CD8^+^ T cells, like their CD4^+^ counterparts, contribute to the induction, progression, and pathogenesis of autoimmune neuroinflammation [2,3]. Myelin-specific CD8^+^ T cells were reported to both aggravate CD4^+^ T cell-mediated experimental autoimmune encephalomyelitis (EAE) [4], an animal model for MS, and to mediate autoimmune CNS disease on their own [5-7]. In particular, adoptively transferred antigen-specific CD8^+^ T cells were found to injure the CNS in models of CD8-mediated EAE [5,6] or in mice that selectively express a neo-self antigen in oligodendrocytes [8,9]. Using continuous confocal imaging, axonal loss observed in these models was shown to result from ‘collateral bystander damage’ by autoaggressive, cytotoxic CD8^+^ T cells, targeting their cognate antigen processed and presented by oligodendrocytes [10]. Histopathological and neurobiological studies in MS also suggest that CD8^+^ T cells hold an active role in disease pathogenesis by targeting oligodendrocytes and the myelin sheath [11].

Due to their ability to function as professional antigen-presenting cells (APCs), CD11c^+^ myeloid DCs play an undisputed role in inciting autoimmunity. In EAE, DCs are critical APCs for the induction of both myelin-specific CD4^+^ and CD8^+^ T cells, and are also a prominent component of CNS-infiltrating cells [12,13]. However, other data indicate that DCs play an important role in initiating tolerance, and that tolerogenic DCs can suppress EAE *in vivo*[14]. Current efforts for the treatment of autoimmune pathogeneses requiring tolerance recovery are focused in the identification of molecules that control the development of tolerogenic DCs [15].

Hepatocyte growth factor (HGF) is a pleiotropic cytokine with potent anti-inflammatory properties shown to act as a potent regulator in multiple animal models of immune-mediated disorders [16-19]. HGF has been shown to govern the development of both human and mouse tolerogenic DCs [20,21]. We recently demonstrated that such a mechanism might account in part for the beneficial action of CNS-restricted overexpression of HGF in myelin oligodendrocyte glycoprotein (MOG)-induced EAE by regulating CD4^+^ T cell-mediated autoimmune responses to MOG [20]. Owing to its strong immunoregulatory and neuroprotective/neurorepair properties [22,23], exogenously supplied HGF was recently further shown to promote recovery in MOG-induced EAE by modulating both the immune response mediated by CD4^+^ T cells and by promoting myelin repair and neural cell development [24]. Because CD8^+^ T cells can further directly mediate motor disability and axon injury in the demyelinated CNS [25] and may actively contribute to neural damage in MS or other CNS inflammatory and degenerative disorders [26], it is important to understand whether HGF could modulate the effector function of antigen-specific CD8^+^ T cells.

To explore the effects of HGF on CD8^+^ T cell functions we used an established *in vitro* model of cytotoxic T-cell-dependent immunity. Our results showed that HGF significantly decreased the generation of effector cytotoxic gp100-petide T cell receptor (Pmel-1) CD8^+^ T cells from naïve splenocytes. HGF greatly reduced the production of inflammatory cytokines and cytolytic enzymes by autoagressive CD8^+^ T cells, including interferon (IFN)-γ, tumor necrosis factor (TNF), perforin, and granzyme B. HGF further diminished the expression of membrane-bound death receptor Fas ligand (FasL), a non-redundant lytic mechanism with cytolytic granule release in cytotoxic T lymphocyte (CTL)-mediated killing. CD8-enriched Pmel-1 splenocytes cultured with HGF demonstrated a considerably lower level of cytolytic activity, as measured by specific killing of antigen-pulsed target cells. Finally, similar results were obtained when HGF-treated CD11c^+^ DCs were cultured with naïve purified Pmel-1 CD8^+^ T cells. These results suggest that HGF reduces CTL responses via professional APCs and may have important implications for CNS inflammatory diseases including MS.

## Methods

### Reagents

Human recombinant HGF (hrHGF) was supplied by T Nakamura (Osaka University, Tokyo, Japan) [27]. Human and murine HGF are cross-reactive. The dose of hrHGF (30 ng/mL) used was chosen based on previous studies analyzing the immunoregulatory effects of hrHGF on CD4^+^ T cell responses [20]. Lower doses were not as effective.

### Cells and cultures

Murine EL-4 lymphoma cells were cultured in DMEM complete medium (CM) supplemented with 10% fetal bovine serum, 50 U/mL penicillin, 50 μg/mL streptomycin and 2 mM L-glutamine (all from Invitrogen/Life Technologies). Splenocytes were derived from Pmel-1 T cell receptor (TCR) transgenic mice, whose TCR recognizes an H-2D^b^-restricted epitope corresponding to amino acids 25–33 of murine and human gp100 (gp100_25-33_), a self-tumor antigen. Approximately 90% of splenic CD3^+^ T cells in Pmel-1 TCR mice are TCRVβ13^+^ CD8^+^ T cells and demonstrate specificity for gp100 [28].

### Purification of mouse splenic CD8^+^ T cells and CD11c^+^ DCs

Mouse splenic CD8^+^ T cells were negatively selected using an anti-mouse CD8^+^ T cell isolation kit (Miltenyi Biotec). To obtain DCs, spleens were minced and incubated with DNase (1 mg/mL) and Liberase HI (Roche) (0.5 mg/mL) for 15 min at room temperature. Cold EDTA was added to a final concentration of 20 mM, and cell suspensions were incubated for 5 min at room temperature before filtering through nylon mesh to remove tissue and cell aggregates. Highly pure mouse splenic DCs were subsequently positively selected using anti-mouse CD11c colloidal superparamagnetic microbeads (Miltenyi Biotec), as reported previously [20]. The purity of CD8^+^ and CD11c^+^ cells, confirmed by flow cytometry, was routinely >95% and >85%, respectively.

### CTL stimulation

Single-cell suspensions of pooled spleens from Pmel-1 mice were prepared by gently homogenizing the tissues and passing them through a 70 μm Nylon cell strainer (Falcon, BD Biosciences). Red blood cells were depleted with ACK lysing buffer (BioWhittaker). Cells were resuspended in DMEM CM with 10 μg/mL gp100_25-33_ peptide (KVPRNQDWL) for 1 h at 37°C. After being washed and counted, hrHGF (30 ng/mL) was added to the cultures every other day starting on day 0. IL-2 (50 U/mL; PeproTech) was added to cultures at days 2 and 4. In certain experiments, purified DCs were treated or not with hrHGF (30 ng/mL) for 24 h at 37°C. After being washed and counted, 2 × 10^6^/mL DCs were resuspended in DMEM CM with 10 μg/mL gp100_25-33_ for 1 h at 37°C. Purified Pmel-1 TCR tg CD8^+^ T cells were mixed with gp100_25-33_-pulsed autologous DCs at a ratio of 10:1 for 5 days. IL-2 (50 U/mL) was added to cultures at days 2 and day 4. Cells cultured for 5 days were tested for functional and phenotypic assays. To evaluate the role of perforin/granzyme-mediated cytotoxicity, the effector cells were pre-treated with 1000 nM concanamycin A (Sigma-Aldrich) for 2 h prior to co-cultures with target cells.

### T cell proliferation assay

For antigen-specific stimulation of mouse CD8^+^ T cells, highly purified DCs treated or not with hrHGF (1–50 ng/mL) for 24 h at 37°C were cultured with gp100_25-33_ (10 μg/mL) and naïve Pmel-1 TCR transgenic CD8^+^ T cells for 5 days. Proliferation was measured by incorporation of ^3^H-methylthymidine (1 μCi/well) during the last 16 h of culture using a filtermate harvester (Packard Instrument Co.) and a 1450 microbeta liquid scintillation counter (PerkinElmer) and was expressed as counts per minute (cpm).

### Immunologic markers and flow cytometry

T cells or DCs were incubated in blocking solution (PBS with 10% FCS) for 20 min on ice prior to staining to block non-specific Fc-mediated interactions, and then stained for 30 min at 4°C with FITC, PE, PerCP-Cy5, or APC fluorochromes conjugated with antibodies (Abs) (1:100) against: CD11c, c-Met, CD8, CD28, CD44, CD62L, CD107a, CTLA-4, Fas-ligand (all Abs from eBioscience), and LFA-1 (Biolegend), or appropriate fluorochrome-conjugated, isotype-matched irrelevant Abs to establish background fluorescence. For intracellular staining of IFN-γ, TNF, perforin, and granzyme B (all Abs from eBioscience), CD8^+^ T cells were stimulated with PMA (1 μg/mL) plus ionomycin (500 ng/mL) in the presence of GolgiStop (10 μg/mL) (BD), and then fixed and permeabilized using BD Cytofix/Cytoperm Plus Kit (BD Biosciences). CD3ζ (eBioscience) staining was evaluated on fixed and permeabilized cells. Live, apoptotic, and dead populations were defined on the basis of 7-AAD Viability Staining Solution from eBioscience according to the manufacturer’s instructions. Samples were processed on a FACS Calibur flow cytometer (Becton Dickinson) and analyzed using FlowJo analysis software (Tree Star, Version 9.3.2).

### Cytotoxicity assay

Functional activities of antigen-specific CTLs were analyzed with a DELFIA® cell cytotoxicity kit (PerkinElmer) according to manufacturer’s instructions. Briefly, target T-lymphocytic leukemia EL-4 cells were pulsed with 10 μg/mL gp100_25-33_ for 1 h at 37°C in DMEM CM, and then washed. EL-4 (1 × 10^5^ cells) were labelled with 50 μM of fluorescence-enhancing ligand bis(acetoxymethyl)2,2′:6′,2′′-terpyridine-t,6′′-dicarboxylate (BATDA) for 30 min at 37°C. After washing, 5 × 10^3^/well labelled cells were mixed with antigen-specific CTLs at the indicated ratio in 96-well plates. Plates were incubated for 4 h at 37 °C. A total of 20 μL of supernatant were harvested from each well and added to wells containing 200 μL of 50 μM Europium solution (Aldrich Chemical) in 0.3 M acetic acid (pH 4). Plates were shaken for 15 min at room temperature and the fluorescence of the Europium-TDA (Eu) chelates formed was quantitated in a time-resolved fluorometer (DELFIA 1234). All assays were performed in triplicates. Spontaneous release was determined as Eu detected in the supernatant of targets incubated in the absence of effector cells. Maximum release was determined as Eu detected in the supernatants of target cells incubated with lysis buffer instead of effectors. Percent specific lysis was calculated according to the formula: (experimental release - spontaneous release)/(maximum release - spontaneous release) × 100.

### Statistical analysis

Statistical comparisons were done using Student’s t-tests. *P* values <0.05 were considered to be statistically significant. All the statistical analyses were performed by GraphPad Prism for Mac, Version 5.0.

## Results

### HGF limits effector Ag-specific CTL generation

**Figure 1:**
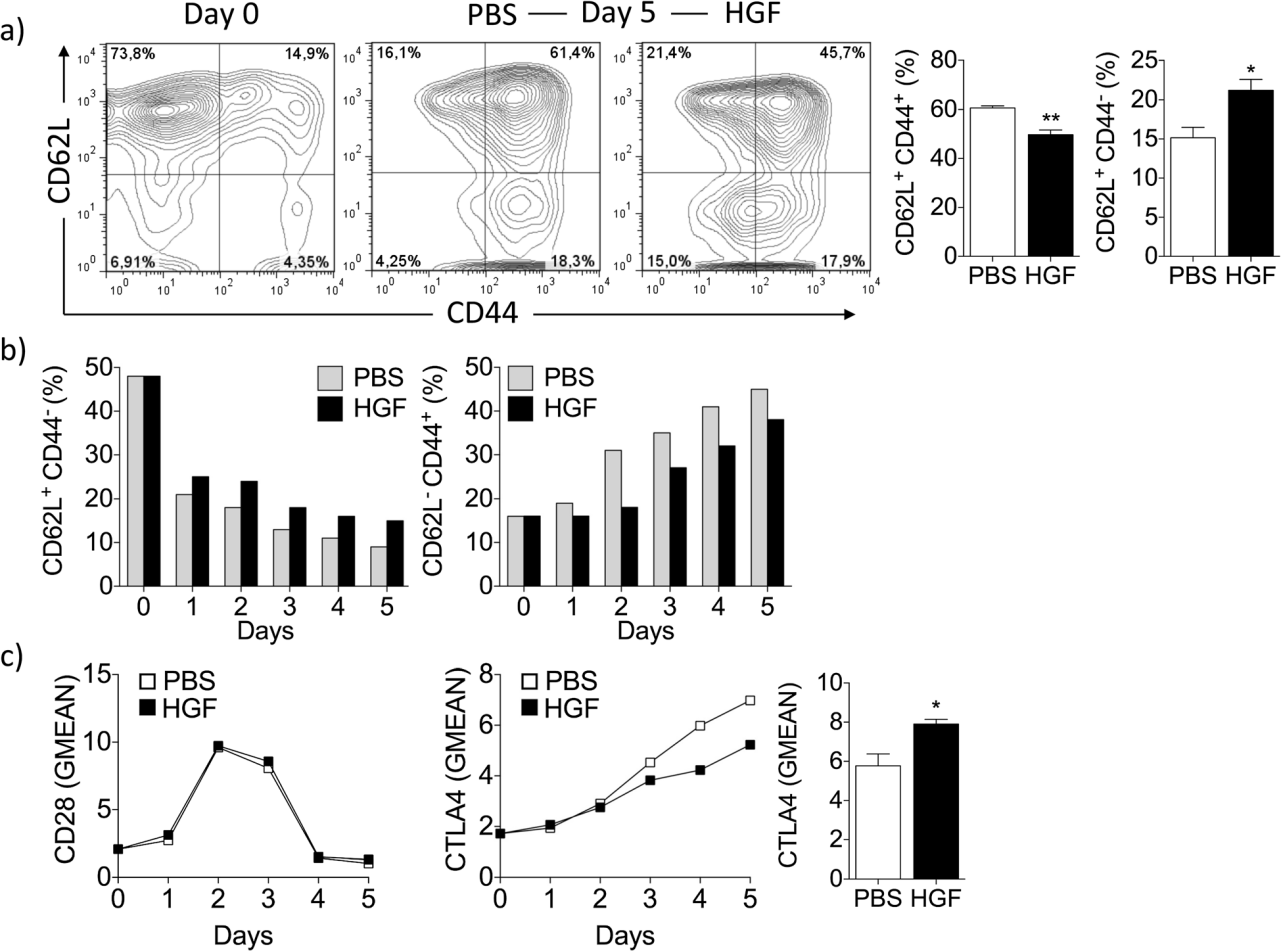
**HGF limits antigen-specific CD8**^**+ **^**T cell activation.** Splenocytes from Pmel-1 transgenic mice were stimulated *in vitro* with gp100_25-33_ (10 μg/mL) for 1 h, followed by addition of IL-2 alone or a combination of IL-2 and HGF cytokines for 5 additional days. **(a)** HGF is effective in maintaining naïve CD62L^hi^CD44^low^ CD8^+^ T cells. T cells were analyzed for the expression of CD44 and CD62L by flow cytometry (*n* = 3 mice per group). At day 0, CD8^+^ T cells showed a naïve CD62L^hi^CD44^low^ phenotype prior to gp100_25-33_ stimulation (a, left panel). At day 5, splenocytes cultured with HGF had a significantly higher percentage of naïve CD62L^hi^CD44^low^ CD8^+^ T cells (a, right panel) than splenocytes cultured in absence of HGF (a, middle panel). Representative contour plots are shown. **(b)** Comparable data were detected on each day for all 5 days tested. **(c)** Flow cytometry analysis of effector cells showed that HGF increased the amount on a per cell basis of CTLA4 starting at day 3 but not CD28 molecules, as indicated by comparative geometric mean of fluorescence (GMEAN) ± SEM of three independent experiments. At day 5, CD8^+^ T cells were analyzed for the expression of CTLA4 by flow cytometry (*n* = 3 mice per group) (right panel). Final GMEAN values are the result of the ratio between the GMEAN obtained with the experimental antibody and the isotype control. *P <0.05 by Student’s *t*-test. Live cells were selected based on gating forward and side scatter. All data were obtained from three independent experiments with similar results.

In order to assess the capacity of HGF to modulate the generation of antigen-specific CD8^+^ T cells, Pmel-1 TCR transgenic splenocytes were stimulated with gp100_25-33_ for 1 h, and then cultured with IL-2 alone or in combination with HGF for 5 days. Five days after antigen stimulation with gp100_25-33_, Pmel-1 splenocyte cultures showed >95% of IL-2 expanded CD8^+^ T cells (Additional file 1: Figure S1a). As shown by 7AAD staining, a similar percentage of antigen-activated Pmel-1 CD8^+^ T cells underwent death 5 days after gp100_25-33_ stimulation when cultured in the absence or presence of HGF (Additional file 1: Figure S1b). These data indicate that HGF has no influence on the viability of the CD8^+^ T cells during their expansion. At day 0, naïve CD8^+^ T cells showed a CD62L^hi^CD44^low^ phenotype prior to gp100_25-33_ stimulation (Figure 1a, left panel). After 5 days of stimulation, splenocyte cultures receiving HGF maintained a significantly higher percentage of naïve CD62L^hi^CD44^low^ CD8^+^ T cells (Figure 1a, right panel) than splenocytes cultured in absence of HGF (Figure 1a, middle panel). Augmented naïve CD62L^high^CD44^low^ phenotype by CD8^+^ T cells was maintained throughout the entire 5 days of effector generation when incubated with HGF (Figure 1b). These results suggest that HGF may inhibit activation of antigen- and cytokine-stimulated T cells and thus may limit the generation of effector and central memory CD8^+^ T cells. HGF supplementation did not decrease the expression of T-cell co-stimulatory molecule CD28 on CD8^+^ T cells (Figure 1c) but was found to upregulate expression of the co-inhibitory receptor cytotoxic T-lymphocyte antigen 4 (CTLA4) by day 3- to 5-cultured CD8^+^ T cells (Figure 1c), a mechanism likely accounting for the apparent reduced effector and central memory CD8^+^ T cell generation over-time.

### HGF limits inflammatory cytokine and cytotoxic effector molecule production by activated CD8^+^ T cells

**Figure 2:**
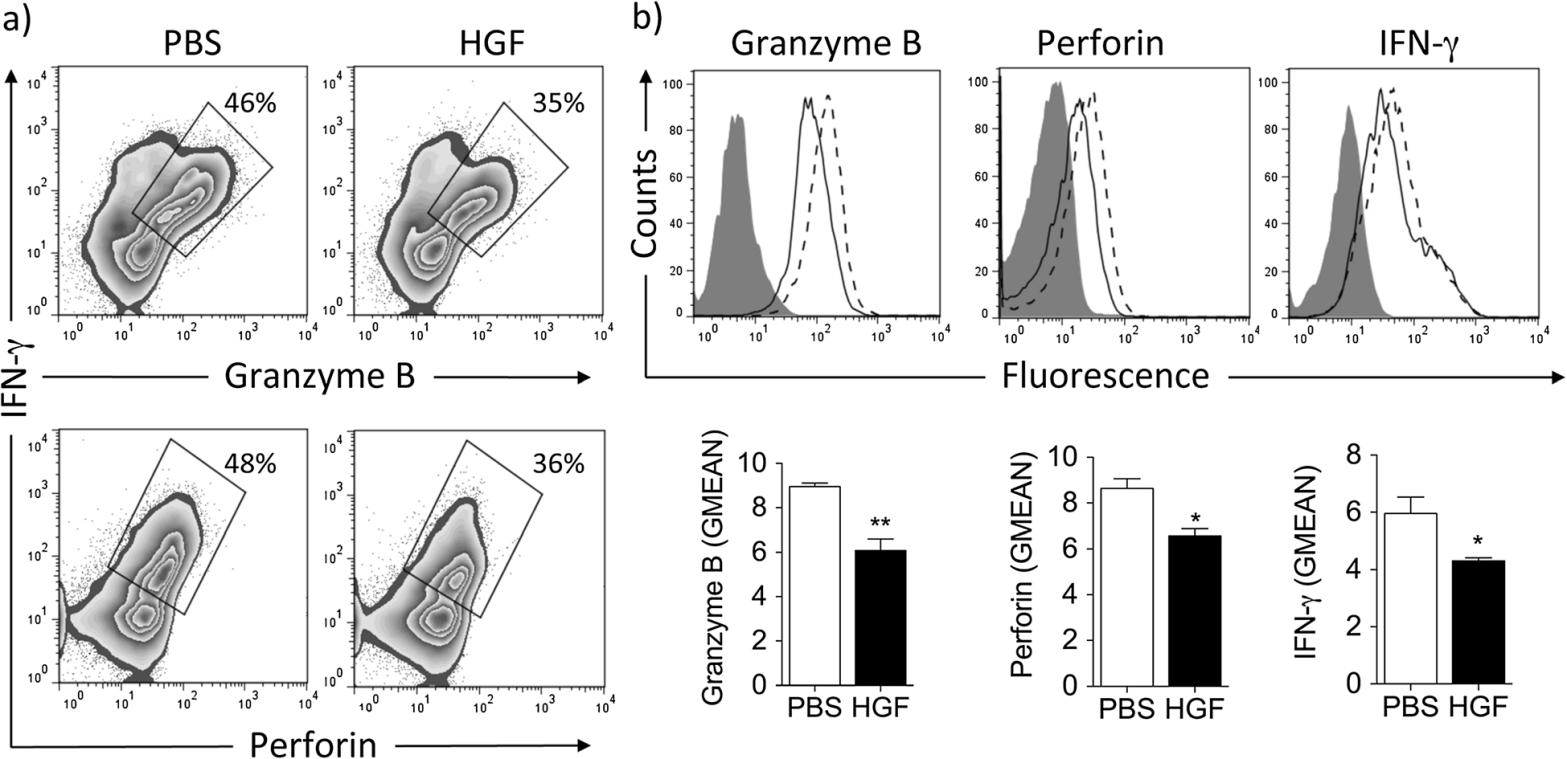
**HGF reduces inflammatory cytokine and cytolytic enzyme production by activated CD8**^**+ **^**T cells.** Pmel-1 splenocytes were cultured for 5 days in IL-2 alone or in combination with HGF, following antigen-specific stimulation. (**a** and **b**) Intracellular cytokine staining of day 5-cultured CD8^+^ T cells showed that addition of HGF not only decreased **(a)** the number of CD8^+^ T cells producing IFN-γ, granzyme B, and perforin but also decreased **(b)** the amount of IFN-γ, granzyme B, and perforin production on a per cell basis, as indicated by comparative geometric mean of fluorescence (GMEAN) ± SEM of three independent experiments. Final GMEAN values are the result of the ratio between the GMEAN obtained with the experimental antibody and the isotype control. Representative contour plots **(a)** and histograms **(b)** are shown. *P <0.05 by Student’s *t*-test. All data were obtained from three independent experiments with similar results.

Since HGF maintained the naïve phenotype of T cells following antigen stimulation, we next examined whether HGF could modulate the effector function of antigen-specific CD8^+^ T cells. Five days following the initiation of Pmel-1 T-cell cultures, we evaluated the expression of indispensable inflammatory cytokines and content of cytotoxic molecules by T cells cultured in IL-2 alone or in combination with HGF, following antigen-specific stimulation. As shown in Figure 2a, addition of HGF decreased the expression of IFN-γ, granzyme B, and perforin. Intracellular cytokine staining of antigen-stimulated day 5-cultured CD8^+^ T cells showed that HGF not only decreased the number of T cells producing IFN-γ, granzyme B, and perforin (Figure 2a), but also decreased the amount of IFN-γ, granzyme B, and perforin production on a per cell basis, as indicated by comparative geometric mean fluorescence intensities (GMEAN) (Figure 2b). Similar flow cytometry profiles were observed 4 h following the initiation of T-cell cultures with gp100_25-33_-pulsed EL-4 cells as targets (Additional file 2: Figure S2a, b). This latter effects could not be attributed to changes in cell size, as CD8^+^ T cells cultured in the presence or absence of HGF maintained the same physical cell size as investigated by forward scatter analyses (Additional file 2: Figure S2c).

### Treatment with HGF reduces the CD8^+^ cytotoxic-T-lymphocyte response

**Figure 3:**
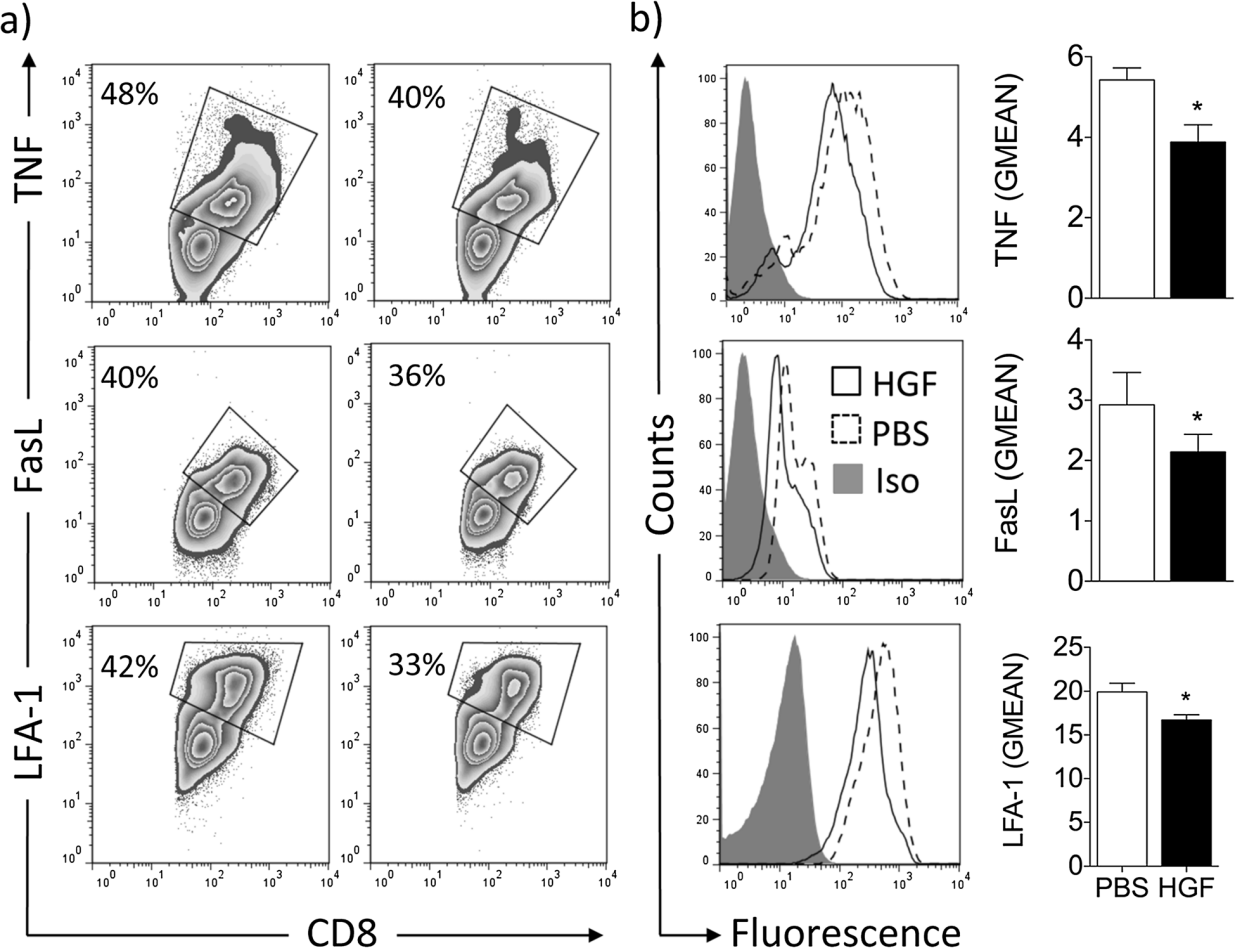
**HGF decreases effector molecules by activated CD8**^**+ **^**T cells.** Splenocytes from Pmel-1 mice were cultured in IL-2 alone or in combination with HGF for 5 days, following antigen-specific stimulation with gp100_25-33_. (**a** and **b**) Flow cytometry analysis of effector cells showed that HGF decreased both **(a)** the number of CD8^+^ T cells expressing TNF, FasL, and LFA-1 and **(b)** the amount on a per cell basis of these molecules that play an important role in CTL cytotoxicity, as indicated by comparative geometric mean of fluorescence (GMEAN) ± SEM of three independent experiments. Final GMEAN values are the result of the ratio between the GMEAN obtained with the experimental antibody and the isotype control. Representative contour plots **(a)** and histograms **(b)** are shown. *P <0.05 by Student’s *t*-test. All data were obtained from three independent experiments with similar results.

Five days following the initiation of Pmel-1 T-cell cultures, we evaluated the expression of other CTL-associated effector molecules by T cells cultured in IL-2 alone or in combination with HGF. HGF treatment was associated with reduced content of granzyme B, perforin, and IFN-γ (Figure 3a, b). In addition, HGF also decreased the production of the Th1 cytokine TNF by antigen-specific CD8^+^ T cells. Moreover, HGF reduced the expression of the death receptor ligand FasL, a non-redundant lytic mechanisms with cytolytic granule release in CTL-mediated killing. Finally, the cell surface molecule lymphocyte function-associated antigen 1 (LFA-1), an important contributor to CTL activation and CTL-mediated direct cell lysis was also significantly reduced. Taken together, these data suggest that HGF significantly reduces both perforin/granzyme B- and Fas-dependent cytotoxicity during an antigen-specific T-cell response. Comparable flow cytometry profiles were detected 4 h following the initiation of T-cell cultures with gp100_25-33_-pulsed EL-4 target cells (Additional file 3: Figure S3a, b). Noticeably, HGF treatment reduced cell surface mobilization of CD107a, a marker commonly used to measure of CTL activity. CD107a is usually found in vesicle membranes, but during CTL-target cell interaction it is mobilized to the cell surface.

### HGF restrains cytotoxicity of antigen-specific CD8^+^ cells

**Figure 4:**
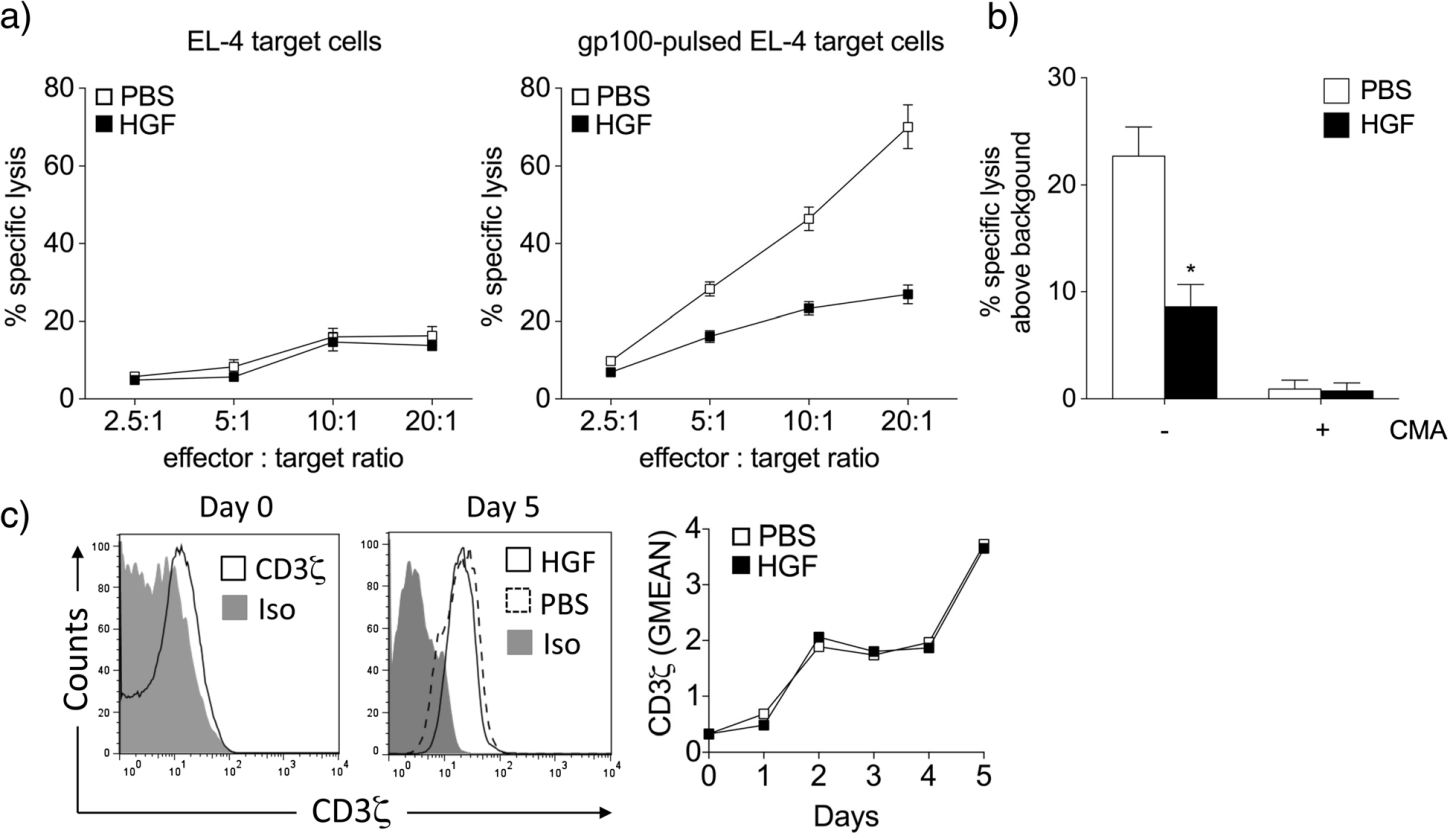
**HGF restrains cytotoxicity of antigen-specific CD8**^**+ **^**cells in a perforin-dependent manner. (a)** The cytolytic capacity of day 5-cultured CD8^+^ T cells was tested using EL-4 cells as targets. CD8^+^ T cells isolated from splenocytes cultured with HGF demonstrated dramatically lower specific cytotoxicity compared to CD8^+^ T cells isolated from splenocytes cultured alone (right panel). gp100_25-33_-stimulated CD8^+^ T cells did not lyse non-pulsed EL-4 target cells (left panel). Results are the mean ± SEM of three independent experiments carried out in triplicates. **(b)** The 4 h cytotoxicity assays (effector : target ratio, 20 : 1) were performed with CTLs pretreated with (+) or without (-) 1000 nM of concanamycin A (ConA) for 2 h. Data are represented as percent of specific lysis above the specific lysis of the EL-4 targets without adding exogenous peptide. A representative experiment of two is shown. *P <0.05 by Student’s *t*-test. **(c)** Flow cytometry analysis of day 5-cultured CD8^+^ T cells showed that addition of HGF did not decrease the level of expression of CD3ζ on a per cell basis, as indicated by comparative geometric mean of fluorescence (GMEAN). Final GMEAN values are the result of the ratio between the GMEAN obtained with the experimental antibody and the isotype control. Representative histograms of day 0 and 5 of three independent experiments with similar results are shown.

Since HGF sustains the naïve activation phenotype of T cells following antigen stimulation and decreases the release of cytolytic granule and pro-inflammatory cytokine mediators and expression of FasL, we next examined whether HGF could modulate the effector function of antigen-specific CD8^+^ T cells. We tested the cytolytic capacity of the cultured T cells using gp100_25-33_-pulsed EL-4 cells as targets. In these experiments, CD8^+^ T cells cultured in HGF demonstrated dramatically reduced specific cytotoxicity compared with CD8^+^ T cells cultured alone (Figure 4a). This decreased cytolytic capacity of CD8^+^ T cells could be explained by a difference in perforin, granzyme B, or LFA-1 expression, as HGF significantly affected expression of all molecules (Figures 2 and 3). We found that concanamycin A, an inhibitor of the perforin-based cytotoxic pathway, almost completely abolished target cell destruction by CTLs (Figure 4b), stressing the importance of this pathway for immediate lytic function mediated by Pmel-1 CD8^+^ T cells. HGF pretreatment did not, however, further reduce the residual killing observed using concanamycin A-treated CTLs. These results argue that HGF mainly limits the cytotoxic, anti-tumor function of Pmel-1 CD8^+^ T cells in a perforin-dependent manner in our experimental settings. Impaired CTL killing was further not associated with changes in CD3ζ expression, a crucial molecule for T-cell activation upon antigen recognition (Figure 4c).

### HGF limits CTL responses by modulating APC functions

**Figure 5:**
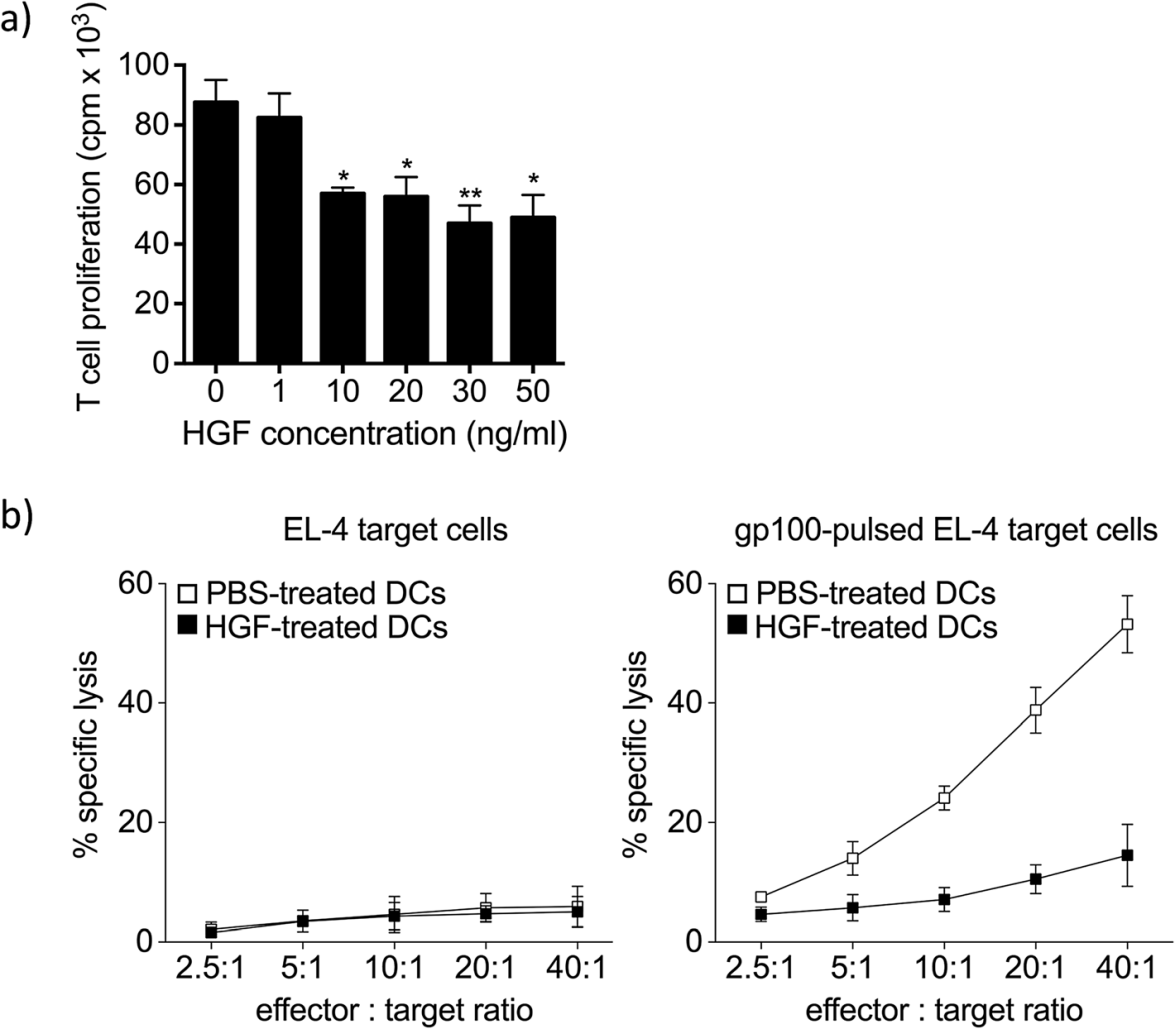
**HGF limits CTL responses by modulating APC functions.** Highly purified CD11c^+^ DCs were cultured for 24 h with either HGF or vehicle and then co-cultured with purified Pmel-1 CD8^+^ T cells in the presence of IL-2 and gp100_25-33_ for 5 days. **(a)** HGF-treated DCs induced low levels of CD8^+^ T-cell proliferation, as measured at day 5 by [^3^H] thymidine incorporation. Values are expressed as counts per minute (cpm). Data are representative of three independent experiments with similar results. *P <0.05; **P <0.01 by Student’s *t*-test. **(b)** CD8^+^ T cells co-cultured with HGF-treated DCs demonstrated dramatically reduced specific cytotoxicity compared with T cells co-cultured with control PBS-treated DCs (right panel). gp100_25-33_-stimulated CD8^+^ T cells did not lyse non-pulsed EL-4 target cells (left panel). Results are the mean ± SEM of three independent experiments carried out in triplicates.

We previously showed that dendritic cells (DCs), required for initiating CTL responses [29], are vulnerable to HGF treatment [20], which can contribute to diminished CTL responses. To address whether this potential mechanism may account for reduced priming of antigen-specific CD8^+^ T cell responses from Pmel-1 splenocyte cultures, we assessed the effect of HGF on the ability of antigen donor DCs cells to prime naïve antigen-specific CD8^+^ T cells. Highly purified CD11c^+^ DCs were cultured for 24 h with either HGF or vehicle and then co-cultured with purified Pmel-1 CD8^+^ T cells in the presence of IL-2 and gp100_25-33_ for 5 days. In contrast to DCs, CD8^+^ T cells did not express the HGF receptor c-Met (Additional file 4: Figure S4). At day 5, CD8^+^ T cell measurement of [^3^H] thymidine incorporation by these cells showed that HGF-treated DCs induced low levels of CD8^+^ T-cell proliferation when compared to those obtained with untreated DCs (Figure 5a). We tested the cytolytic capacity of the CD8^+^ T cells co-cultured in the presence of HGF- or vehicle-treated DCs using gp100_25-33_-pulsed EL-4 target cells. As shown in Figure 5b, CD8^+^ T cells co-cultured with HGF-treated DCs demonstrated dramatically lower specific cytotoxicity compared to T cells co-cultured with control DCs. As expected, CTLs generated in the presence of HGF-treated DCS were associated with reduced expression of cytolytic markers, such as perforin, granzyme B, and FasL. Altogether, these results indicate that HGF acts directly on DCs by reducing their ability to both generate and prime antigen-specific CD8^+^ T cell responses.

## Discussion

HGF is a multifunctional cytokine that blunts inflammation in a variety of inflammatory T-cell-mediated disease models, suggesting that HGF suppresses a common inflammatory process. In MOG-induced EAE, a common model of MS primarily mediated by encephalitogenic CD4^+^ T cell responses and characterized by demyelination and axonal loss [30], we have previously demonstrated that overexpression of neuronal HGF attenuated disease progression in part via anti-inflammatory signals [20]. Using this MS model, we established that HGF exerts an anti-inflammatory effect through the generation of tolerogenic DCs and the subsequent suppression of autoreactive peripheral Th1 and Th17 cells, leading to reduced CD4^+^ T cell-mediated CNS injury. Whether HGF modulates cell-mediated immunity driven by MHC class I-restricted CD8^+^ T cells remained unknown.

In addition to pathogenic CD4^+^ T cells, multiple observations support the idea that CD8^+^ T cells are involved in pathogenesis of CNS autoimmunity, as active contributors to the development of neuroinflammation. In MS, CD8^+^ T cells outnumber by far CD4^+^ T cells in both acute and chronic inflammatory lesions. In addition, while CD4^+^ T cells show a primarily perivascular distribution, CD8^+^ T cells can be detected in the parenchyma [31,32]. Although normally poorly expressed, MHC class I molecules are highly expressed within the MS lesion on astrocytes, oligodendrocytes, and neurons, suggesting that CD8^+^ T cells could be directly engaging these cell types [33-36]. Using an established model of murine CTL-mediated killing we have here examined whether HGF could regulate autoaggressive, cytotoxic CD8^+^ T cell responses.

In this study, we found that HGF treatment of DCs reduced the generation and functions of cytotoxic effector CD8^+^ T lymphocytes and subsequent MHC class I-restricted CTL-mediated cytolysis of target cells. The development of naïve cytotoxic CD8^+^ T cells into CTLs requires specific recognition of antigen:MHC class I complexes on professional APCs in conjunction with co-stimulatory signals. Secondary recognition of antigen:MHC class I complexes on a target cell by a CTL leads to the death of the target cell. Our findings indicate that HGF treatment interfered with the development of autoagressive CTLs and not their capacity to recognize their target cells. In particular, we found that HGF treatment increased the levels of the inhibitory counter-receptor CTLA4 molecules expressed on CD8^+^ T cells but did not affect the expression of the CD3ζ molecules. CTLA4-mediated negative co-stimulation together with other regulatory mechanisms mediated by tolerogenic APCs likely accounts for maintenance of high frequencies of naïve CTL precursors incapable of cytotoxicity in splenocyte cultures supplemented with HGF.

CTLs mediate the killing of target cells via two major pathways, a granule-dependent (perforin/granzyme B) and independent (FasL induced cell death) mechanism. Here we found that HGF treatment decreased the levels of the effector CTL molecules IFN-γ, TNF, perforin, and granzyme B as well as the expression of CD107a, a marker of CD8^+^ T-cell degranulation following stimulation. Using a potent inhibitor of the perforin-based cytotoxic pathway, concanamycin A, we found that HGF potently inhibits CTL-mediated killing through interference with the granule exocytosis pathway. Our data further revealed that HGF treatment reduced CTL-bound FasL expression on CD8^+^ T cells, suggestive of an action of HGF on the dual perforin/granzyme B- and Fas-based CTL-mediated cytotoxicity. As both the perforin/granzyme B-dependent granule exocytosis pathway [37-41] and the Fas signaling [42,43] have been implicated as potential mechanisms in oligodendrocyte and/or axonal injury and demyelination in MS, our findings taken together suggest that HGF might be effective in a potential therapeutic approach to reduce CTL effector function in CTL-mediated human autoimmune disorder of the CNS.

## Conclusions

Altogether, our findings indicate that HGF treatment limits both the generation and effector functions of CTLs. Complementary to its impact on CD4^+^ T-cell CNS autoimmunity, our findings further suggest that HGF treatment could be exploited to control CD8^+^ T-cell-mediated, MHC I-restricted autoimmune dysfunctions such as MS. By coupling immunosuppressive properties on both CD4^+^ and CD8^+^ T cell effector responses and neurorepair actions, HGF appears thus to be a promising candidate for the treatment of inflammatory demyelinating neurodegenerative diseases such as MS. One must, however, point out that such observations are preliminary, and do not establish the safety of HGF administration over the long term, which may include potential adverse events. In particular, additional research is warranted to evaluate the impact of HGF therapy in anti-tumor immunity as the potent immune inhibition exerted by HGF may help tumor cells to escape from immune surveillance.

## Competing interests

The authors declare no competing financial interests.

## Authors’ contributions

MB, NM, and PHL designed research, analyzed data, and wrote the paper; PW analyzed data, gave conceptual advice, and discussed the results at all stages; MB and GS performed the experiments; PW provided the Pmel-1 mice; and PHL supervised the study. All authors read, commented, and approved the final manuscript.

## Supplementary Material

Additional file 1: Figure S1 HGF does not affect *in vitro*-expanded CD8^+^ T cell viability. (**a**) Five days after stimulation with gp100_25-33_, Pmel-1 splenocyte cultures showed >95% of IL-2 expanded CD8^+^ T cells. (**b**) A similar percentage of antigen-activated Pmel-1 CD8^+^ T cells underwent death 5 days after gp100_25-33_ stimulation when cultured in the absence or presence of HGF, as shown by 7AAD staining. Representative contour plots are shown. All data were obtained from three independent experiments with similar results. Click here for file

Additional file 2: Figure S2 HGF restrains CTL effector molecule expression. *In vitro*-expanded CD8^+^ T cell were cultured for 4 h with gp100_25-33_-pulsed EL-4 target cells. (**a** and **b**) Intracellular cytokine staining of CD8^+^ T cells showed that addition of HGF to Pmel-1 splenocyte cultures not only decreased (**a**) the number of CD8^+^ T cells producing IFN-γ, granzyme B, and perforin but also decreased (**b**) the amount of IFN-γ, granzyme B, and perforin production on a per cell basis, as indicated by comparative geometric mean of fluorescence. (**c**) Forward scatter analyses of CD8^+^ T cells indicate that HGF supplementation did not affect cell size. Data are presented overlapping the control analysis. Representative contour plots (**a**) and histograms (**b, c**) are shown. All data were obtained from three independent experiments with similar results. Click here for file

Additional file 3: Figure S3 HGF dampens effector molecules by activated CD8^+^ T cells. *In vitro*-expanded CD8^+^ T cell were cultured for 4 h with gp100_25-33_-pulsed EL-4 target cells. (**a** and **b**) Flow cytometry analysis of effector cells showed that addition of HGF to Pmel-1 splenocyte cultures decreased both (**a**) the number of CD8^+^ T cells expressing CD107, TNF, FasL, and LFA-1 and (**b**) the amount on a per cell basis of these molecules that play an important role in CTL cytotoxicity, as indicated by comparative geometric mean of fluorescence. Representative contour plots (**a**) and histograms (**b**) are shown. All data were obtained from three independent experiments with similar results. Click here for file

Additional file 4: Figure S4 DCs but not CD8^+^ T cells show cell-surface expression of the HGF receptor c-Met. Expression of c-Met at the cell surface of CD11c^+^ DCs and CD8^+^ T cells was examined by flow cytometry. Illustrative histograms depict the expression of c-Met (open histograms) and control staining with isotype-matched antibody (shaded histograms). Click here for file

## References

[B1] McFarland HF Martin R Multiple sclerosis: a complicated picture of autoimmunity Nat Immunol 2007 10 913 919 10.1038/ni1507 17712344

[B2] Mars LT Saikali P Liblau RS Arbour N Contribution of CD8 T lymphocytes to the immuno-pathogenesis of multiple sclerosis and its animal models Biochim Biophys Acta 2011 10 151 161 10.1016/j.bbadis.2010.07.006 20637863 PMC5052066

[B3] Zozulya AL Wiendl H The role of CD8 suppressors versus destructors in autoimmune central nervous system inflammation Hum Immunol 2008 10 797 804 10.1016/j.humimm.2008.07.014 18723060

[B4] Mars LT Bauer J Gross DA Bucciarelli F Firat H Hudrisier D Lemonnier F Kosmatopoulos K Liblau RS CD8 T cell responses to myelin oligodendrocyte glycoprotein-derived peptides in humanized HLA-A*0201-transgenic mice J Immunol 2007 10 5090 5098 17911594 10.4049/jimmunol.179.8.5090

[B5] Huseby ES Liggitt D Brabb T Schnabel B Ohlen C Goverman J A pathogenic role for myelin-specific CD8(+) T cells in a model for multiple sclerosis J Exp Med 2001 10 669 676 10.1084/jem.194.5.669 11535634 PMC2195947

[B6] Sun D Whitaker JN Huang Z Liu D Coleclough C Wekerle H Raine CS Myelin antigen-specific CD8+ T cells are encephalitogenic and produce severe disease in C57BL/6 mice J Immunol 2001 10 7579 7587 11390514 10.4049/jimmunol.166.12.7579

[B7] Friese MA Jakobsen KB Friis L Etzensperger R Craner MJ McMahon RM Jensen LT Huygelen V Jones EY Bell JI Fugger L Opposing effects of HLA class I molecules in tuning autoreactive CD8+ T cells in multiple sclerosis Nat Med 2008 10 1227 1235 10.1038/nm.1881 18953350

[B8] Na SY Cao Y Toben C Nitschke L Stadelmann C Gold R Schimpl A Hunig T Naive CD8 T-cells initiate spontaneous autoimmunity to a sequestered model antigen of the central nervous system Brain 2008 10 2353 2365 10.1093/brain/awn148 18669487

[B9] Saxena A Bauer J Scheikl T Zappulla J Audebert M Desbois S Waisman A Lassmann H Liblau RS Mars LT Cutting edge: multiple sclerosis-like lesions induced by effector CD8 T cells recognizing a sequestered antigen on oligodendrocytes J Immunol 2008 10 1617 1621 18641296 10.4049/jimmunol.181.3.1617

[B10] Sobottka B Harrer MD Ziegler U Fischer K Wiendl H Hunig T Becher B Goebels N Collateral bystander damage by myelin-directed CD8+ T cells causes axonal loss Am J Pathol 2009 10 1160 1166 10.2353/ajpath.2009.090340 19700745 PMC2731134

[B11] Friese MA Fugger L Autoreactive CD8+ T cells in multiple sclerosis: a new target for therapy? Brain 2005 10 1747 1763 10.1093/brain/awh578 15975943

[B12] Bailey SL Schreiner B McMahon EJ Miller SD CNS myeloid DCs presenting endogenous myelin peptides ’preferentially’ polarize CD4+ T(H)-17 cells in relapsing EAE Nat Immunol 2007 10 172 180 10.1038/ni1430 17206145

[B13] Ji Q Castelli L Goverman JM MHC class I-restricted myelin epitopes are cross-presented by Tip-DCs that promote determinant spreading to CD8(+) T cells Nat Immunol 2013 10 254 261 10.1038/ni.2513 23291597 PMC3581685

[B14] Li H Zhang GX Chen Y Xu H Fitzgerald DC Zhao Z Rostami A CD11c + CD11b + dendritic cells play an important role in intravenous tolerance and the suppression of experimental autoimmune encephalomyelitis J Immunol 2008 10 2483 2493 18684939 10.4049/jimmunol.181.4.2483 PMC2676731

[B15] Pulendran B Tang H Manicassamy S Programming dendritic cells to induce T(H)2 and tolerogenic responses Nat Immunol 2010 10 647 655 10.1038/ni.1894 20644570

[B16] Okunishi K Dohi M Nakagome K Tanaka R Mizuno S Matsumoto K Miyazaki J Nakamura T Yamamoto K A novel role of hepatocyte growth factor as an immune regulator through suppressing dendritic cell function J Immunol 2005 10 4745 4753 16177122 10.4049/jimmunol.175.7.4745

[B17] Futamatsu H Suzuki J Mizuno S Koga N Adachi S Kosuge H Maejima Y Hirao K Nakamura T Isobe M Hepatocyte growth factor ameliorates the progression of experimental autoimmune myocarditis: a potential role for induction of T helper 2 cytokines Circ Res 2005 10 823 830 10.1161/01.RES.0000163016.52653.2e 15774858

[B18] Kuroiwa T Iwasaki T Imado T Sekiguchi M Fujimoto J Sano H Hepatocyte growth factor prevents lupus nephritis in a murine lupus model of chronic graft-versus-host disease Arthritis Res Ther 2006 10 R123 10.1186/ar2012 16859527 PMC1779408

[B19] Okunishi K Dohi M Fujio K Nakagome K Tabata Y Okasora T Seki M Shibuya M Imamura M Harada H Tanaka R Yamamoto K Hepatocyte growth factor significantly suppresses collagen-induced arthritis in mice J Immunol 2007 10 5504 5513 17911637 10.4049/jimmunol.179.8.5504

[B20] Benkhoucha M Santiago-Raber ML Schneiter G Chofflon M Funakoshi H Nakamura T Lalive PH Hepatocyte growth factor inhibits CNS autoimmunity by inducing tolerogenic dendritic cells and CD25 + Foxp3+ regulatory T cells Proc Natl Acad Sci USA 2010 10 6424 6429 10.1073/pnas.0912437107 20332205 PMC2851995

[B21] Rutella S Bonanno G Procoli A Mariotti A de Ritis DG Curti A Danese S Pessina G Pandolfi S Natoni F Di Febo A Scambia G Manfredini R Salati S Ferrari S Pierelli L Leone G Lemoli RM Hepatocyte growth factor favors monocyte differentiation into regulatory interleukin (IL)-10++IL-12low/neg accessory cells with dendritic-cell features Blood 2006 10 218 227 10.1182/blood-2005-08-3141 16527888

[B22] Lalive PH Paglinawan R Biollaz G Kappos EA Leone DP Malipiero U Relvas JB Moransard M Suter T Fontana A TGF-beta-treated microglia induce oligodendrocyte precursor cell chemotaxis through the HGF-c-Met pathway Eur J Immunol 2005 10 727 737 10.1002/eji.200425430 15724248

[B23] Molnarfi N Benkhoucha M Bjarnadottir K Juillard C Lalive PH Interferon-beta induces hepatocyte growth factor in monocytes of multiple sclerosis patients PLoS One 2012 10 e49882 10.1371/journal.pone.0049882 23166786 PMC3498184

[B24] Bai L Lennon DP Caplan AI DeChant A Hecker J Kranso J Zaremba A Miller RH Hepatocyte growth factor mediates mesenchymal stem cell-induced recovery in multiple sclerosis models Nat Neurosci 2012 10 862 879 10.1038/nn.3109 22610068 PMC3427471

[B25] Deb C Lafrance-Corey RG Schmalstieg WF Sauer BM Wang H German CL Windebank AJ Rodriguez M Howe CL CD8+ T cells cause disability and axon loss in a mouse model of multiple sclerosis PLoS One 2010 10 e12478 10.1371/journal.pone.0012478 20814579 PMC2930011

[B26] Melzer N Meuth SG Wiendl H CD8+ T cells and neuronal damage: direct and collateral mechanisms of cytotoxicity and impaired electrical excitability FASEB J 2009 10 3659 3673 10.1096/fj.09-136200 19567369

[B27] Nakamura T Nishizawa T Hagiya M Seki T Shimonishi M Sugimura A Tashiro K Shimizu S Molecular cloning and expression of human hepatocyte growth factor Nature 1989 10 440 443 10.1038/342440a0 2531289

[B28] Overwijk WW Theoret MR Finkelstein SE Surman DR de Jong LA Vyth-Dreese FA Dellemijn TA Antony PA Spiess PJ Palmer DC Heimann DM Klebanoff CA Yu Z Hwang LN Feigenbaum L Kruisbeek AM Rosenberg SA Restifo NP Tumor regression and autoimmunity after reversal of a functionally tolerant state of self-reactive CD8+ T cells J Exp Med 2003 10 569 580 10.1084/jem.20030590 12925674 PMC2194177

[B29] Villadangos JA Heath WR Carbone FR Outside looking in: the inner workings of the cross-presentation pathway within dendritic cells Trends Immunol 2007 10 45 47 10.1016/j.it.2006.12.008 17197240

[B30] Zamvil SS Steinman L The T lymphocyte in experimental allergic encephalomyelitis Ann Rev Immunol 1990 10 579 621 10.1146/annurev.iy.08.040190.003051 2188675

[B31] Booss J Esiri MM Tourtellotte WW Mason DY Immunohistological analysis of T lymphocyte subsets in the central nervous system in chronic progressive multiple sclerosis J Neurol Sci 1983 10 219 232 10.1016/0022-510X(83)90201-0 6607973

[B32] Hauser SL Bhan AK Gilles F Kemp M Kerr C Weiner HL Immunohistochemical analysis of the cellular infiltrate in multiple sclerosis lesions Ann Neurol 1986 10 578 587 10.1002/ana.410190610 3524414

[B33] Traugott U Reinherz EL Raine CS Multiple sclerosis: distribution of T cell subsets and Ia-positive macrophages in lesions of different ages J Neuroimmunol 1983 10 201 221 10.1016/0165-5728(83)90036-X 6222066

[B34] Wong GH Bartlett PF Clark-Lewis I Battye F Schrader JW Inducible expression of H-2 and Ia antigens on brain cells Nature 1984 10 688 691 10.1038/310688a0 6433204

[B35] Neumann H Cavalie A Jenne DE Wekerle H Induction of MHC class I genes in neurons Science 1995 10 549 552 10.1126/science.7624779 7624779

[B36] Neumann H Schmidt H Cavalie A Jenne D Wekerle H Major histocompatibility complex (MHC) class I gene expression in single neurons of the central nervous system: differential regulation by interferon (IFN)-gamma and tumor necrosis factor (TNF)-alpha J Exp Med 1997 10 305 316 10.1084/jem.185.2.305 9016879 PMC2196130

[B37] Jacobsen M Cepok S Quak E Happel M Gaber R Ziegler A Schock S Oertel WH Sommer N Hemmer B Oligoclonal expansion of memory CD8+ T cells in cerebrospinal fluid from multiple sclerosis patients Brain 2002 10 538 550 10.1093/brain/awf059 11872611

[B38] Junker A Ivanidze J Malotka J Eiglmeier I Lassmann H Wekerle H Meinl E Hohlfeld R Dornmair K Multiple sclerosis: T-cell receptor expression in distinct brain regions Brain 2007 10 2789 2799 10.1093/brain/awm214 17890278

[B39] Neumann H Medana IM Bauer J Lassmann H Cytotoxic T lymphocytes in autoimmune and degenerative CNS diseases Trends Neurosci 2002 10 313 319 10.1016/S0166-2236(02)02154-9 12086750

[B40] Serafini B Rosicarelli B Magliozzi R Stigliano E Capello E Mancardi GL Aloisi F Dendritic cells in multiple sclerosis lesions: maturation stage, myelin uptake, and interaction with proliferating T cells J Neuropathol Exp Neurol 2006 10 124 141 16462204 10.1097/01.jnen.0000199572.96472.1c

[B41] Lassmann H Bruck W Lucchinetti CF The immunopathology of multiple sclerosis: an overview Brain Pathol 2007 10 210 218 10.1111/j.1750-3639.2007.00064.x 17388952 PMC8095582

[B42] D’Souza SD Bonetti B Balasingam V Cashman NR Barker PA Troutt AB Raine CS Antel JP Multiple sclerosis: Fas signaling in oligodendrocyte cell death J Exp Med 1996 10 2361 2370 10.1084/jem.184.6.2361 8976190 PMC2196365

[B43] Dowling P Husar W Menonna J Donnenfeld H Cook S Sidhu M Cell death and birth in multiple sclerosis brain J Neurol Sci 1997 10 1 11 10.1016/S0022-510X(97)05213-1 9168159

