# Supplementary figures and images for: The neurotrophic hepatocyte growth factor attenuates CD8+ cytotoxic T-lymphocyte activity
Source: J Neuroinflammation. 2013 Dec 17;10:154. doi: 10.1186/1742-2094-10-154 (PMC3881506; doi:10.1186/1742-2094-10-154)

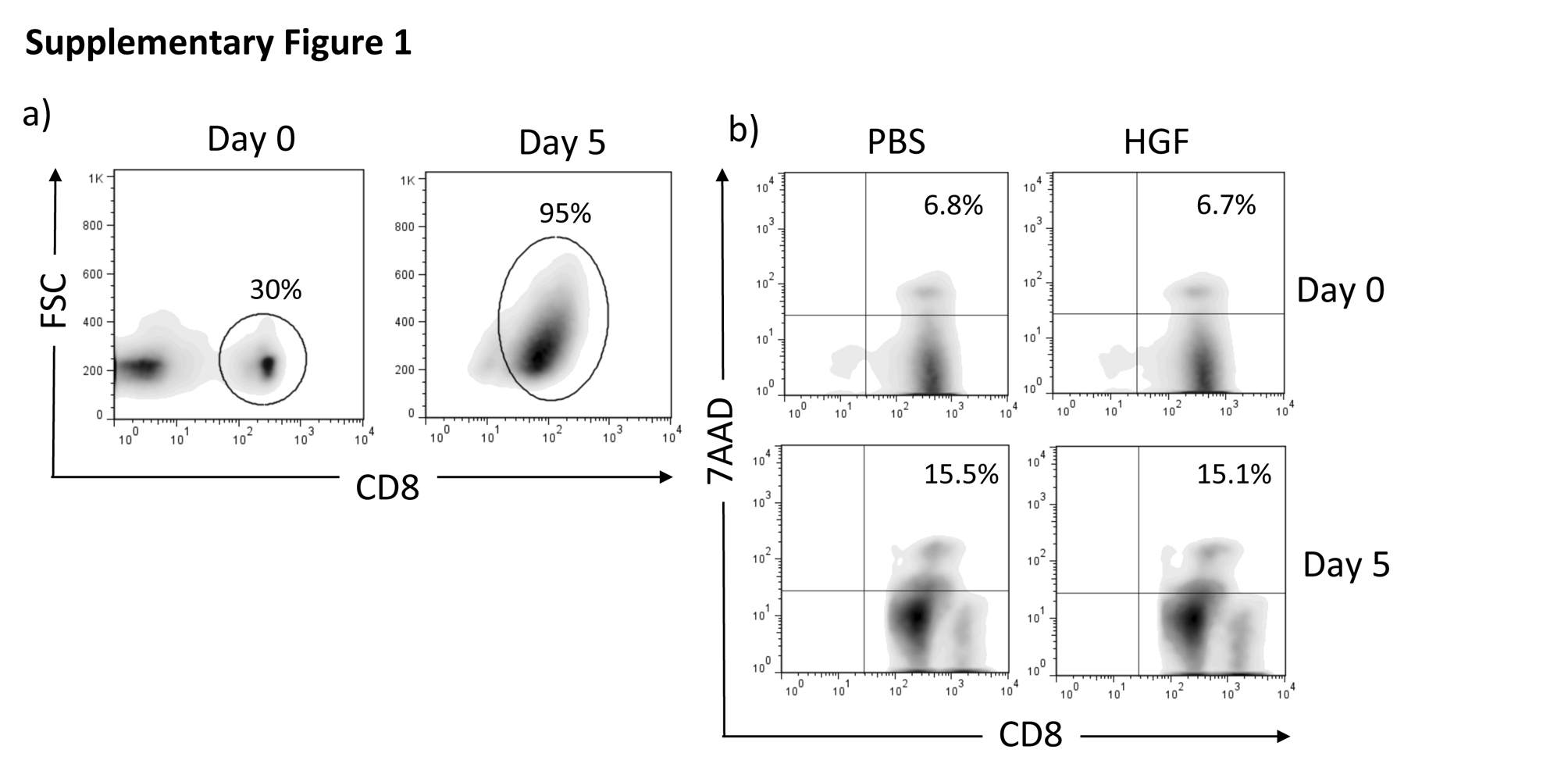

Supplement: Additional file 1: Figure S1 — HGF does not affect in vitro-expanded CD8+ T cell viability. (a) Five days after stimulation with gp10025-33, Pmel-1 splenocyte cultures showed >95% of IL-2 expanded CD8+ T cells. (b) A similar percentage of antigen-activated Pmel-1 CD8+ T cells underwent death 5 days after gp10025-33 stimulation when cultured in the absence or presence of HGF, as shown by 7AAD staining. Representative contour plots are shown. All data were obtained from three independent experiments with similar results. [file 1742-2094-10-154-S1.tiff]

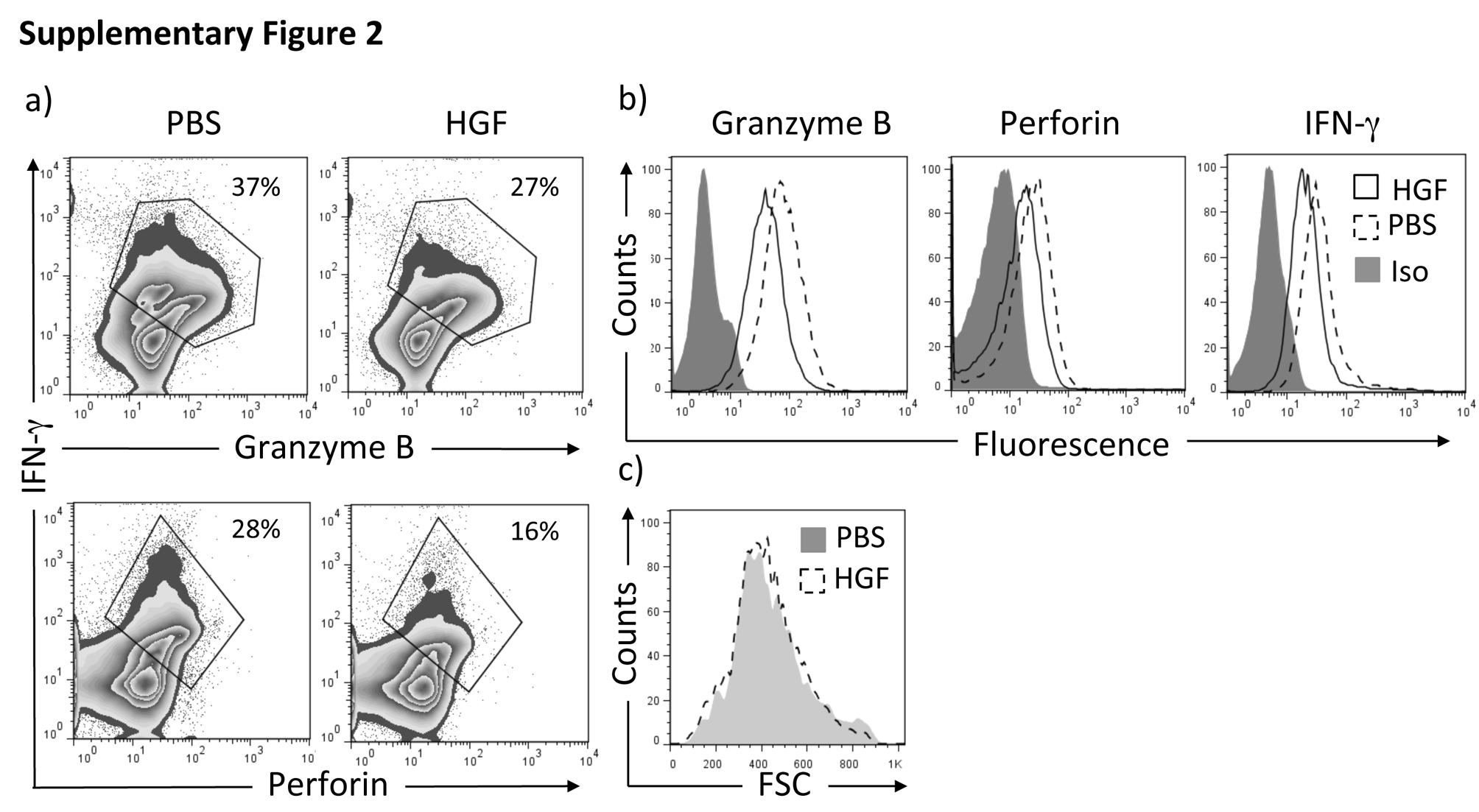

Supplement: Additional file 2: Figure S2 — HGF restrains CTL effector molecule expression. In vitro-expanded CD8+ T cell were cultured for 4 h with gp10025-33-pulsed EL-4 target cells. (a and b) Intracellular cytokine staining of CD8+ T cells showed that addition of HGF to Pmel-1 splenocyte cultures not only decreased (a) the number of CD8+ T cells producing IFN-γ, granzyme B, and perforin but also decreased (b) the amount of IFN-γ, granzyme B, and perforin production on a per cell basis, as indicated by comparative geometric mean of fluorescence. (c) Forward scatter analyses of CD8+ T cells indicate that HGF supplementation did not affect cell size. Data are presented overlapping the control analysis. Representative contour plots (a) and histograms (b, c) are shown. All data were obtained from three independent experiments with similar results. [file 1742-2094-10-154-S2.tiff]

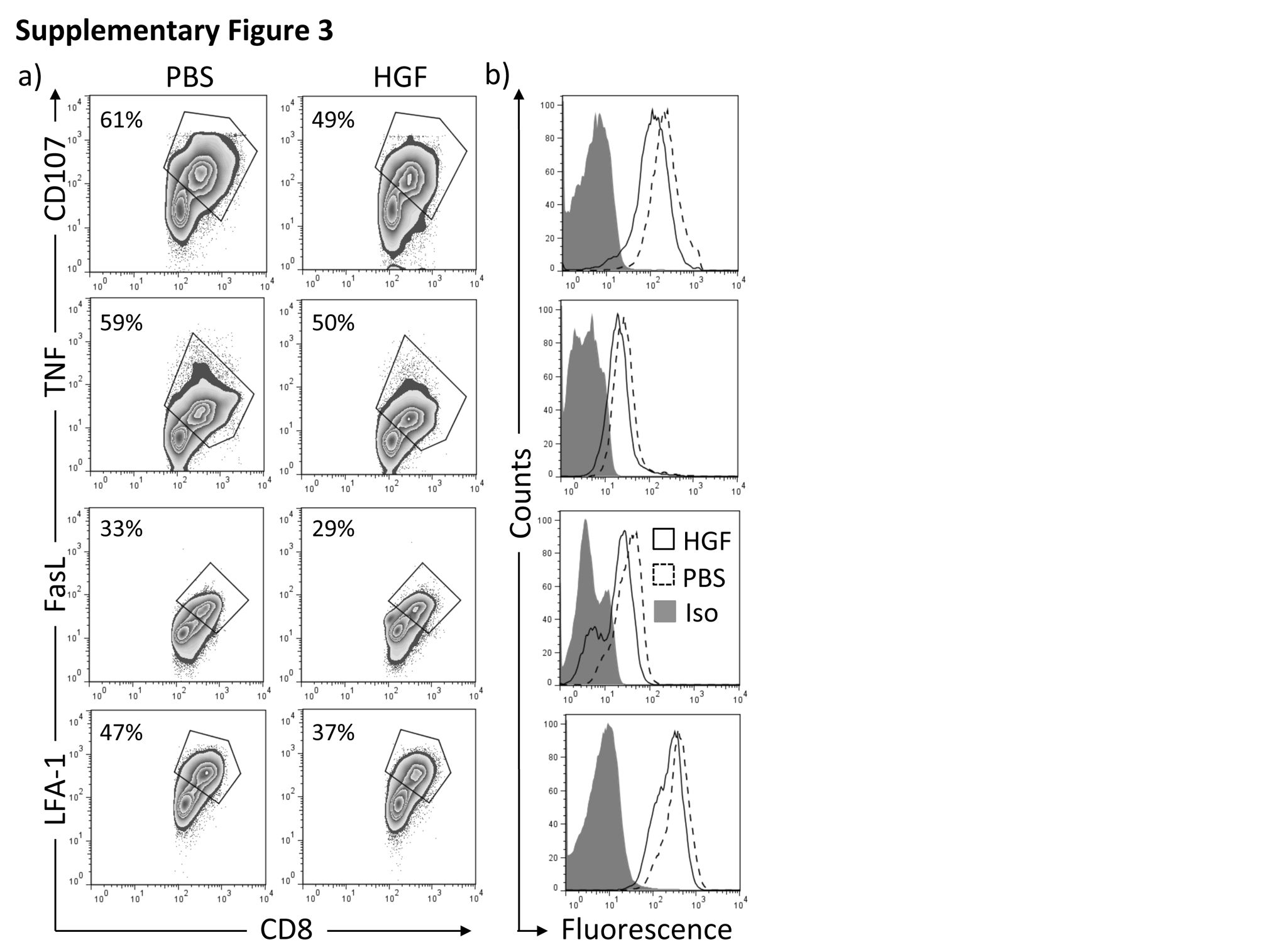

Supplement: Additional file 3: Figure S3 — HGF dampens effector molecules by activated CD8+ T cells. In vitro-expanded CD8+ T cell were cultured for 4 h with gp10025-33-pulsed EL-4 target cells. (a and b) Flow cytometry analysis of effector cells showed that addition of HGF to Pmel-1 splenocyte cultures decreased both (a) the number of CD8+ T cells expressing CD107, TNF, FasL, and LFA-1 and (b) the amount on a per cell basis of these molecules that play an important role in CTL cytotoxicity, as indicated by comparative geometric mean of fluorescence. Representative contour plots (a) and histograms (b) are shown. All data were obtained from three independent experiments with similar results. [file 1742-2094-10-154-S3.tiff]

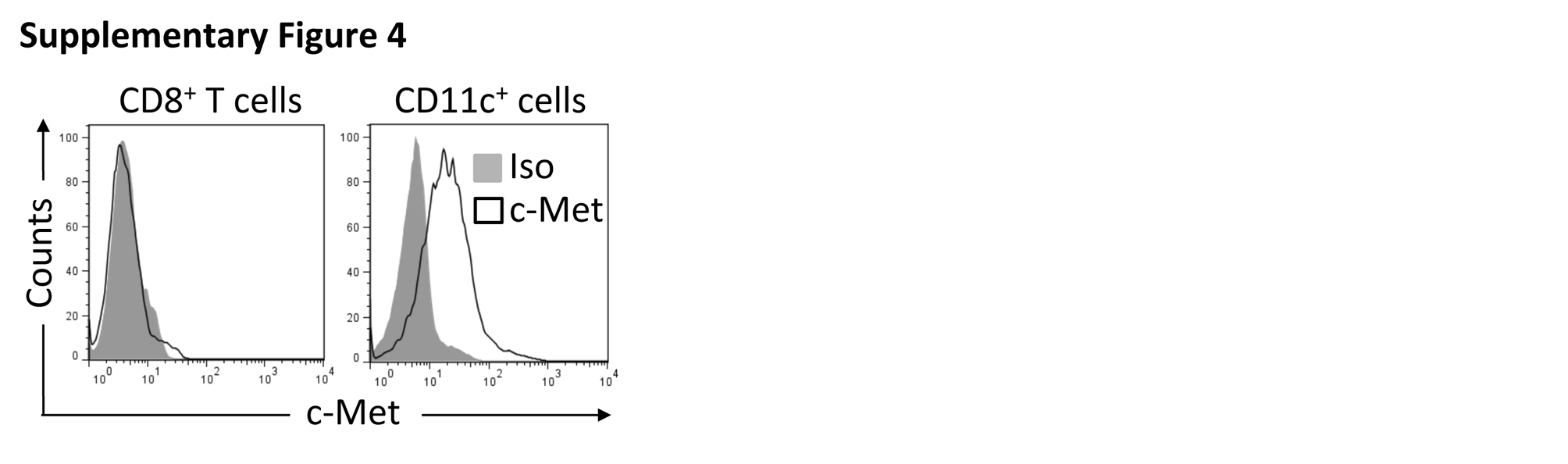

Supplement: Additional file 4: Figure S4 — DCs but not CD8+ T cells show cell-surface expression of the HGF receptor c-Met. Expression of c-Met at the cell surface of CD11c+ DCs and CD8+ T cells was examined by flow cytometry. Illustrative histograms depict the expression of c-Met (open histograms) and control staining with isotype-matched antibody (shaded histograms). [file 1742-2094-10-154-S4.tiff]
